# DNA Damage, n-3 Long-Chain PUFA Levels and Proteomic Profile in Brazilian Children and Adolescents

**DOI:** 10.3390/nu13082483

**Published:** 2021-07-21

**Authors:** Tamiris Trevisan de Barros, Vinicius de Paula Venancio, Lívia Cristina Hernandes, Lusania Maria Greggi Antunes, Elaine Hillesheim, Roberta Garcia Salomão, Mariana Giaretta Mathias, Carolina Almeida Coelho-Landell, Roseli Borges Donegá Toffano, Maria Olimpia Ribeiro do Vale Almada, José Simon Camelo-Junior, Sofia Moco, Ornella Cominetti, Fábio da Veiga Ued, Jim Kaput, Jacqueline Pontes Monteiro

**Affiliations:** 1 Department of Pediatrics, Medical School of Ribeirao Preto, University of Sao Paulo, Sao Paulo 14049-900, Brazil; elainehillesheim@usp.br (E.H.); rsalomao@usp.br (R.G.S.); marimathias@hotmail.com (M.G.M.); k_rolacoelho@hotmail.com (C.A.C.-L.); roselibdt@hotmail.com (R.B.D.T.); madovale@yahoo.com.br (M.O.R.d.V.A.); jscamelo@fmrp.usp.br (J.S.C.-J.); jacque@fmrp.usp.br (J.P.M.); 2 School of Pharmaceutical Sciences of Ribeirao Preto, University of Sao Paulo, Sao Paulo 14049-900, Brazil; venancio.vinicius@alumni.usp.br (V.d.P.V.); lusania@fcfrp.usp.br (L.M.G.A.); 3 Nestlé Research, Société des Produits Nestlé SA, EPFL Innovation Park, CH1015 Lausanne, Switzerland; s.moco@vu.nl (S.M.); ornella.cominetti@rd.nestle.com (O.C.); jkaput@gmail.com (J.K.); 4 Department of Health Sciences, Medical School of Ribeirao Preto, University of Sao Paulo, Sao Paulo 14049-900, Brazil; uedfabio@gmail.com

**Keywords:** DNA damage, proteomic, fatty acids, child, adolescents

## Abstract

Fatty acids play a significant role in maintaining cellular and DNA protection and we previously found an inverse relationship between blood levels of eicosapentaenoic acid (EPA) and docosahexaenoic acid (DHA) and DNA damage. The aim of this study was to explore differences in proteomic profiles, for 117 pro-inflammatory proteins, in two previously defined groups of individuals with different DNA damage and EPA and DHA levels. Healthy children and adolescents (*n* = 140) aged 9 to 13 years old in an urban area of Brazil were divided by k-means cluster test into two clusters of DNA damage (tail intensity) using the comet assay (cluster 1 = 5.9% ± 1.2 and cluster 2 = 13.8% ± 3.1) in our previous study. The cluster with higher DNA damage and lower levels of DHA (6.2 ± 1.6 mg/dL; 5.4 ± 1.3 mg/dL, *p* = 0.003) and EPA (0.6 ± 0.2 mg/dL; 0.5 ± 0.1 mg/dL, *p* < 0.001) presented increased expression of the proteins CDK8–CCNC, PIK3CA–PIK3R1, KYNU, and PRKCB, which are involved in pro-inflammatory pathways. Our findings support the hypothesis that low levels of n-3 long-chain PUFA may have a less protective role against DNA damage through expression of pro-inflammatory proteins, such as CDK8–CCNC, PIK3CA–PIK3R1, KYNU, and PRKCB.

## 1. Introduction

Recent advances in omics sciences have enabled the study of health processes that facilitate the development of personalized medicine [1,2,3]. These methodological improvements are also advancing precision nutrition based on the availability of big data in genomics, metabolomics, proteomics, and various computational analyses. A major emphasis of these approaches is the identification and validation of biomarkers that assess molecular processes that can be altered through nutrition [3].

Nutrients, alone or in combinations, can influence several mechanisms of biological systems, including the expression of proteins [4]. Proteomics studies can help elucidate the interactions between nutrients and gene expression, leading to a better understanding of the role of nutrition on maintenance of health [4]. 

DNA damage is caused by various factors that are intrinsic to cell metabolism, such as reactive oxygen species, or by external stressors such as sunlight or free radicals generated from environmental pollutants. Pathological processes induced by oxidative stress, inflammatory processes, or cellular degeneration may result in atherosclerosis, neurodegenerative diseases, and malignant diseases, associated with increased DNA damage [5,6,7,8,9].

In response to internal and external stressors, DNA damage responses are initiated for maintaining normal cellular functions [5,7,8,9]. These processes depend on regulation of the cell cycle, a complex regulatory system that involves changes in amount and modifications of DNA-damage-inducible proteins [5,7]. The DNA damage response is initiated with the recognition of an injury by sensor complexes such as the Mre11–Rad50–Nbs1 (MRN) mediator complex and the proliferating cell nuclear antigen (PCNA)-related Rad9–Rad1–Hus1 complex [10,11]. Kinases and cyclin-dependent kinases are also involved with DNA damage. These regulatory cell cycle proteins help to regulate gene transcription, acting also as modulators of DNA damage response [12,13]. Other physiological systems also respond to these internal and external stressors: increased levels of pro-inflammatory proteins such as nuclear factor kappa B (NF-κB), toll-like receptor 4 (TLR4), and several interleukins also contribute to processes involved in DNA damage [14]. Proteomic analysis may be used to evaluate the factors involved in mechanisms of DNA damage.

Fatty acids play a significant role in maintaining cellular and DNA protection [15]. Our previous study demonstrated an inverse relationship between blood levels of long-chain n-3 polyunsaturated fatty acids (PUFAs)—eicosapentaenoic acid (EPA) and docosahexaenoic acid (DHA)—and DNA damage [16].

The anti-inflammatory properties of EPA and DHA are thought to help protect against DNA damage through the synthesis of mediators to solve the inflammation process [17,18,19] and via competition with the conversion of n-6 fatty acids to arachidonic acid (AA), which reduces the formation of pro-inflammatory AA-derived eicosanoids [20]. In addition, evidence suggests that these long-chain omega-3 PUFAs participate in modulating cell cycle phases related to induction of apoptosis [21].

Given the potential interactions between components of the inflammatory systems and DNA damage, we hypothesized that individuals with greater DNA damage and lower DHA and EPA levels may have distinct proteomic profiles among the 117 pro-inflammatory proteins tested by our methods compared with individuals with lower DNA damage and higher DHA and EPA levels. Hence, the aim of this study was to test whether proteomic expression differs in two groups previously described in the study of Barros et al. [16], defined by contrasting levels of DNA damage and by DHA and EPA red blood cells levels. 

## 2. Materials and Methods

### 2.1. Study Design and Population

This cross-sectional study is part of a micronutrient intervention project previously reported [22]. The trial was registered on ClinicalTrials.gov (NCT01823744), accessed on 19 July 2021. Subjects agreed to participate by signing a statement of informed assent, and a parent signed an informed consent. Brazilian National Ethics Committee approved this study (CONEP 00969412.6.0000.5440) [22].

In brief, healthy children and adolescents aged from 9 to 13 years, 11 months and 29 days old were recruited in 2013 from one private and two public schools located in a neighborhood in the city of Ribeirão Preto (São Paulo, Brazil) [22]. All subjects have access to electricity, sanitation, potable water, and internet. The municipal human development index (MHDP) is 0.8, which is considered very high [22]. Exclusion criteria included dietary restrictions, including weight-loss interventions, current intake of vitamin or mineral supplement, chronic diseases, at least three episodes of liquid stools within the 24 hours preceding data collection, at least one episode of axillary temperature higher than 37 °C within the 15 days before the assessment, and participation in another clinical trial within the 4 weeks prior to the study. Clinical condition and sexual maturity rate were assessed by a pediatrician using Tanner criteria [23]. All physiological assessments were performed by trained personnel and included weight and height measurements and blood collection. Nutritional status was obtained by the classification of body mass index (BMI) in accordance to World Health Organization (WHO) [24]. 

### 2.2. Fatty Acids Assessment 

The polyunsaturated fatty acids, EPA and DHA, assessed in red blood cells (200 µL RBC lysed with 200 µL of NH_4_Cl 82.29 mg/mL, NaHCO_3_ 10.00 mg/mL, and EDTA 292.2 mg/mL), were analyzed by gas–liquid chromatography as the method described by Masood et al [16,25,26].

### 2.3. DNA Damage Assessment

DNA damage quantification was obtained by alkaline single-cell gel electrophoresis (comet assay) as the methodology described by Singh et al. [27] and Tice et al. [28], with minor modifications [16]. Freshly collected blood (25 µL) preserved in EDTA was added to 0.5% (*m/v*) low-melting-point agarose (270 µL) at 37 °C (Invitrogen, Carlsbad, CA, USA) [16]. An amount of 80 µL of the cell suspension was transferred onto microscope slides already covered with 1.5% (*m/v*) normal-melting-point agarose (Invitrogen, Carlsbad, CA, USA) [16]. Slides were maintained at 4 °C for 20 min for agarose solidification. The slides were then submerged in lysis solution (2.5 M NaCl; 100 mM EDTA, Sigma-Aldrich, St Louis, MO, USA; 10 mM Tris, Sigma-Aldrich, St Louis, MO, USA; 10 % (*v/v*) DMSO, Sigma-Aldrich, St Louis, MO, USA; 1% (*v/v*) Triton X-100, pH 10) and maintained protected from light at 4 °C for 24 h [16]. Slides were submerged in electrophoresis solution (300 mM NaOH, 1 mM EDTA, pH > 13) for 20 min at 4 °C, and then placed in a horizontal electrophoresis unit with the same solution [16]. Electrophoresis was performed for 20 min at 0.87 V/cm (25 V and 300 mA). During the electrophoresis, fragments of damaged DNA migrate through the slides, creating a tail shape [16]. Slides were then washed in a neutralization buffer (0.4 M Tris pH 7.5, Sigma-Aldrich, St Louis, MO, USA) at 4 °C for 5 min and then fixed in ethanol for 2 min [16]. The slides were stained with 200 µL of GelRED^TM^ solution (Biotium, Fremont, CA, USA, 1:1000) for 3 min and then covered with coverslips, right before the analysis [16]. A total of 100 nucleoids per subject were randomly chosen and analyzed with a fluorescence microscope (filter 516–560 nm, barrier filter 590 nm, 40 × objective) [16]. Samples were assessed using the software CometAssay IV (Perceptive Instruments Ltd.), through which we obtained tail intensity values, by the measurement of emitted light in the formed tail of nucleoid, linearly related to DNA damage [29].

### 2.4. Proteomic Analysis

One hundred and seventeen plasma proteins involved in regulating inflammation were selected from the 1129 quantified proteins by the SomaLogic proteomic Somascan assay version 1® (SomaLogic Inc., 2945 Wilderness Place, Boulder, CO 80301, USA) (Supplementary Table S1). Somascan consists of DNA Aptamers, which are similar in concept and function to monoclonal antibodies. In brief, the proteins to be measured bind to their cognate nucleic acid-based SOMAmers (modified aptamers), which include a photo-cleavable biotin (PCB) and a fluorescent label at the 5’end. Streptavidin-coated beads (SA) capture the bound protein–SOMAmer complexes by the PCB present on the SOMAmer. Unbound aptamers are washed away, and the bound proteins are tagged with biotin. UV light cleaves the PCB, releasing protein–SOMAmer complexes into solution. SOMAmers are quantified by hybridization. Each probe is proportional to the amount of SOMAmer recovered, quantifying the amount of protein present in the original sample [30,31]. Laboratory analysis and quantification were conducted by SomaLogic.

### 2.5. Statistical Analyses

All statistical analyses were performed using SPSS® 20.0. We previously found the K = 2 clusters presenting different degrees of DNA damage [16] using k-means cluster tests. Analysis of covariance (ANCOVA) was performed to compare proteomic analysis between these clusters, adjusting for the confounding variables age, sex, body mass index (BMI), and energy intake. We also performed multiple regression analysis to evaluate the strength of the relationship between the variables.

## 3. Results

Over 151 children and adolescents were recruited for the study, 141 individuals met the inclusion criteria and 140 had DNA damage results that met our predetermined quality standard. Two clusters were previously identified differing in DNA damage using k-means cluster test: cluster 1 (*n* = 62) had a tail intensity of 5.9% ± 1.2 and cluster 2 (*n* = 78) had a tail intensity of 13.8% ± 3.1 [16]. The characteristics of the two groups of DNA damage regarding age, sex, and sexual maturity rate were not significantly different (Table 1). Sixty-eight individuals (48.6%) were overweight or obese children and adolescents, without difference between groups.

**Figure 1:**
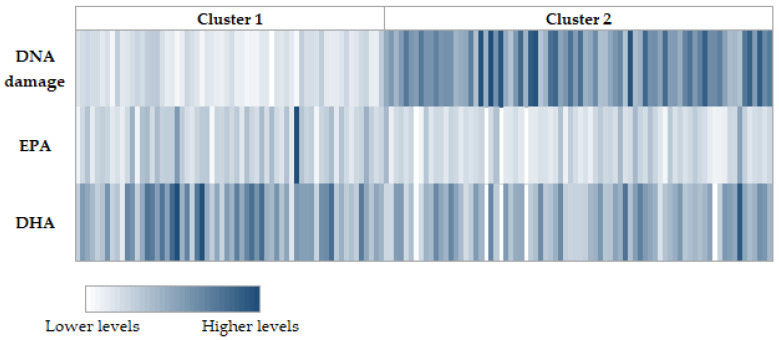
Heatmap representing association between DNA damage, long-chain PUFAs, and sample clusters.

As shown in our previous article [16], levels of the long-chain PUFA, DHA and EPA, were greater in cluster 1 (lower DNA damage), compared with cluster 2 (Table 1; Figure 1).

Of 117 plasma proteins analyzed (Supplementary Table S1), 6 were statistically different between the two clusters: cyclin dependent kinase 8 (CDK8), cyclin C (CCNC), kynureninase (KYNU), phosphatidylinositol 3-kinase catalytic subunit alpha (PIK3CA), phosphatidylinositol 3-kinase regulatory subunit 1 (PIK3R1), and protein kinase C beta (PRKCB) (Table 2). Levels of CDK8–CCNC, PIK3CA–PIK3R1, KYNU, and PRKCB were lower in the cluster with lower DNA damage (cluster 1) with higher levels of DHA and EPA. Detailed information about these proteins is described in Table 3.

A statistically significant relationship was found between these proteins and DNA damage using multiple regression analysis. The model with all proteins and confounding variables explained 13% of the variation in DNA damage, after being adjusted (Table 4). Regression analysis models were also performed with each protein separately (Table 4), and the relationships remained statistically significant.

**Figure 2:**
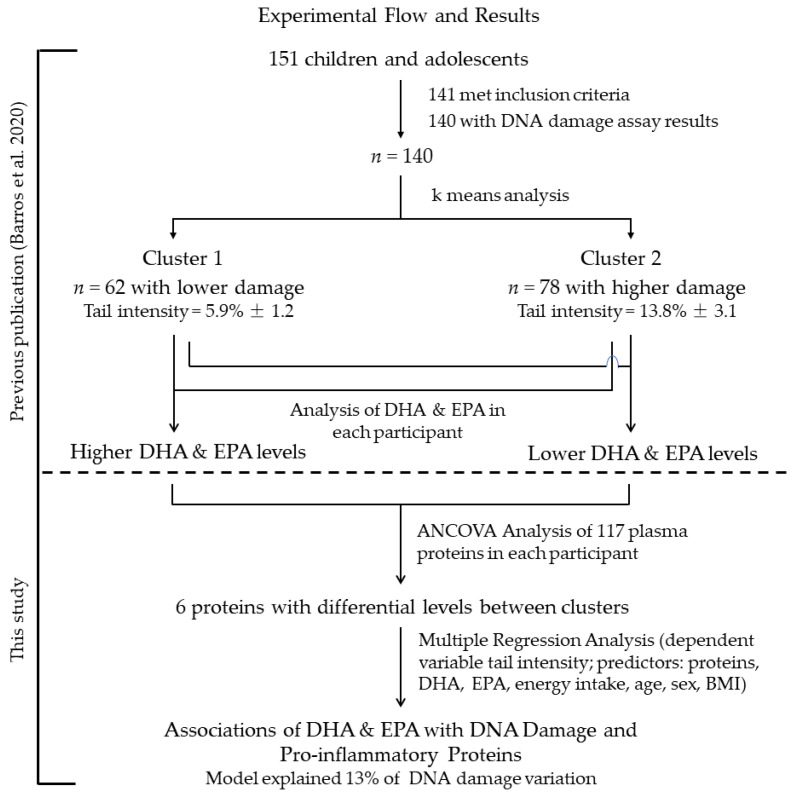
Experimental flow and main results.

Figure 2 illustrates the experimental flow of the present study and our first published one regarding the DNA damage, DHA and EPA clusters [16], and the main results found in this study.

## 4. Discussion

Seemingly separate biological pathways often interact in the functioning of the biological system. For example, our previous work found differences in levels of DHA and EPA and the amount of DNA damage [16]. In this study, we found associations between DHA and EPA, DNA damage, and six proteins involved in inflammatory responses. Inflammatory responses are often initiated by reactive oxygen species produced by cell metabolism, or by ionizing radiation, chemical agents, and UVB light, all of which can lead to inflammation and DNA damage [45]. The interacting responses activate several signaling pathways, leading to the upregulation of protein expression. Moreover, the overexpression of certain proteins can further stimulate ROS production [45].

Cyclins and CDKs participate in cell cycle regulation but with different substrates and functions. The transcription-associated CDKs, including CDK7, CDK8, CDK9, CDK12, and CDK13, are critical regulators of gene expression [12]. Evidence suggests several novel functions of these CDKs, including regulation of epigenetic modifications, intronic polyadenylation, DNA damage responses, and genomic stability [12]. Cyclin-dependent kinase 8 (CDK8) is a transcription-regulating serine/threonine kinase and a subunit of CDK module [32,36]. CDK8 is a mediator kinase and operates as a positive regulator of transcription, acting in the phosphorylation of polymerase II in specific networks, including cancer signaling pathways [32,36,46]. However, CDK8 also functions in the pathogenesis of cancer as a mediator of damage-induced tumor-promoting paracrine activities [46]. The tumor suppressor gene, p53, plays a crucial role in cell cycle control and DNA damage and is positively regulated by CDK8 [33]. Under stress conditions inducing DNA damage, p53 acts to block the cell cycle, thereby allowing DNA repair or cellular apoptosis [47,48]. An increased expression of CDK8 occurs in some types of cancer, while DNA damage is closely linked to the etiology of malignant diseases. Failure in repair is associated with mutagenesis and the generation of cells with genetic instability. 

In the present study, higher levels of CDK8–CCNC complex were identified in the cluster of higher DNA damage and lower DHA and EPA levels (cluster 2). Since the individuals of the present study are healthy and present no type of cancer, it seems that CDK8–CCNC may act as components of the normal cell repair machinery, because CDK8 also phosphorylates several other proteins, many of which are related to DNA repair and transcription [12,49]. Cyclin C (CCNC), also a component of the repair machinery, is the regulatory subunit of CDK8 [50]. Stieg et al showed that CCNC also plays a role in response to oxidative stress and induces apoptosis [38,51]. Others have found that CDK8–CCNC may play a role in lipid metabolism and are key repressors of lipogenic gene expression, de novo lipogenesis, and lipid accumulation in mammals [52]. This function of CDK8–CCNC occurs through site-specific phosphorylation of the nuclear SREBP-1c protein, resulting in rapid degradation of this central regulator of lipid metabolism [52]. Further studies are needed to specifically relate DHA, EPA, CDK8–CCNC, and DNA damage.

PIK3CA and PIK3R1 are, respectively, a catalytic and a regulatory subunit of phosphatidylinositol 3-kinase (PI3K), a cell membrane signaling component that acts in a variety of cellular functions, such as survival, growth, metabolism, and chemotaxis [53,54]. Increased ROS levels activate the PI3K/Akt and cause a reduction in autophagy occurrence, leading to proteins and DNA damage [55,56,57]. In our findings, PIK3CA and PIK3R1 were significantly higher in the cluster of children with higher DNA damage and lower DHA and EPA levels (cluster 2). MRE11–RAD50–NBS1 (MRN) mediator complex is increased in DNA damage and functions by recognizing double-strand breaks. The NBS1 subunit of MRN interacts with PI3K, stimulating its activity, which could explain its higher levels in cluster 2 [10]. Other authors found similar associations. Chandra Pal et al. showed a reduction in UVB-induced inflammation and DNA damage through the inhibition PI3K signaling [58]. Nguyen et al. found that the inhibition of PI3K/AKT pathway can lead to a reduction in telomeric DNA damage through the modulation of CD4 T cells [59].

The cluster with lower DNA damage (cluster 1) had higher levels of DHA and EPA in red blood cells. Denys et al. showed that DHA and EPA replace arachidonic acid (ARA) in the composition of membrane phospholipids and suppress IL-2 gene expression by inhibiting the membrane recruitment of protein kinase C (PKC) and blocking the nuclear translocation of NF-kB involved in T cell proliferation, interrupting the inflammatory pathway [60,61], which can reduce DNA damage. Kim et al. demonstrated that treatment with DHA led to a reduction in PI3K in lung cancer cells [62]. That cell culture study also showed that addition of DHA induced autophagy by downregulation of the PI3K/AKT signaling pathway [62]. Apoptosis is a mechanism regulated by the cell cycle in order to protect cells against DNA damage propagation, preventing the proliferation of cells with injured DNA [5,6,7,8]. Increased apoptosis could explain the increased levels of these intracellular proteins in the plasma of the participants in our study.

A recent study demonstrated that the PI3K pathway is inhibited by the downregulation of kynureninase (KYNU), which corroborates another finding of our study [40]. KYNU catalyzes the degradation of its metabolite kynurenine, produced in the first step of tryptophan catabolism. Under conditions of inflammation, KYNU levels are elevated [39,63,64]. DNA damage caused by ROS may increase the de novo synthesis of nicotinamide adenine dinucleotide (NAD) by kynurenine pathway. Poly ADP-ribose polymerase (PARP) uses NAD produced by this pathway, leading to the depletion of NAD. Increased DNA damage may stimulate this pathway and deplete NAD stores, thereby inducing cell dysfunction or cell death [45,65,66]. Gomes et al. triggered inflammation through the administration of lipopolysaccharide (LPS) to mice, resulting in an increase in kynurenine and tryptophan. The increase of kynurenine and tryptophan was attenuated by EPA and DHA treatment, which also contributed to the decrease of inflammatory biomarkers [67]. These results may explain our findings regarding the increased level of KYNU in the group of higher DNA damage and lower levels of n-3 PUFA (cluster 2) compared with the cluster 1, demonstrating a potential protective effect of DHA and EPA against DNA damage mediated in part by the kynurenine pathway.

Protein kinase C β (PRKCB) was also increased in individuals with higher DNA damage. ROS production is stimulated by PRKCB, thus leading to an increase in DNA damage, which can explain the association found in this study [68,69]. Others have demonstrated that PRKCB regulates cellular signaling pathways involved in the autophagic process [43]. Patergnani et al. found that the activation of this protein leads to inhibition of autophagy, suggesting a negative regulation of this pathway. The downregulation of apoptosis pathways may cause persistence of injuries to the genome through proliferation of cells with damaged DNA [43]. Davidson et al. showed that a diet rich in n-3 PUFA and pectin led to a reduced expression of PRKCB and enhanced apoptosis in mice colon cancer cells in comparison to an n-6 PUFA/pectin diet, demonstrating a protective effect by suppression of PRKCB expression [70].

This study has some limitations, such as the lack of an additional biomarker to assess DNA damage. DNA damage was measured in lymphocytes (white blood cells) and cannot be considered a biomarker of DNA damage for other cells in the whole body. However, the comet assay is a sensitive method for the assessment of DNA damage and has been widely used. In addition, our study population is composed of healthy children and adolescents, and DNA damage (tail intensity values) is considered low [71]. Reproducibility of these results is appropriate in other medically compromised populations.

In conclusion, these findings show that children and adolescents with higher DNA damage and lower levels of n-3 PUFAs—DHA and EPA—presented higher levels of proteins involved in inflammatory mechanisms. The importance of our study lies in the long-term negative impact of these pro-inflammatory proteins on DNA damage recurrence in children and adolescents with low DHA and EPA levels. DNA damage is a crucial component in the development of several diseases [5,6,7,8]. Malignant and neurodegenerative pathologies are recognized by higher rates of DNA damage in association with reduced levels of repair or autophagy, which causes a persistence of injuries in gene structure and promotes the proliferation of abnormal cells, characteristics in these diseases [5,6,7,8,43,45]. Further longitudinal studies are needed to validate the expression of proteins and omega-3 PUFA levels in a small cohort.

## Figures and Tables

**Table 1:** Characterization of DNA damage clusters *.

Characteristics	Cluster 1 (*n* = 62)	Cluster 2 (*n* = 78)	*p*-Value
Age (years old)	11.4 ± 1.1	11.4 ± 1.1	0.989 ^3^
Sex (*n*; %)			0.728 ^4^
Male	26 (41.9)	35 (44.9)	
Female	36 (58.1)	43 (55.1)	
Sexual Maturity Rate (*n*; %) ^1^			0.497 ^4^
1	4 (6.4)	10 (12.8)	
2	29 (46.8)	32 (41.0)	
3	21 (33.9)	27 (34.6)	
4	5 (8.1)	8 (10.3)	
5	3 (4.8)	1 (1.3)	
Nutritional status (*n*; %) ^2^			0.332 ^4^
Eutrophic	29 (46.8)	43 (55.1)	
Overweight	19 (30.6)	14 (17.9)	
Obese	14 (22.6)	21 (26.9)	
Energy Intake (kcal/day)	2061.6 ± 664.5	2065.2 ± 848.6	0.978 ^3^
Tail intensity (%)	5.9 ± 1.2	13.8 ± 3.1	<0.001
DHA (mg/dL)	6.2 ± 1.6	5.4 ± 1.3	0.003 ^3^
EPA (mg/dL)	0.6 ± 0.2	0.5 ± 0.1	<0.001 ^3^

* Table adapted from Barros TT et al. (2020). Cluster 1, lower DNA damage, and cluster 2, higher DNA damage. ^1^ Sexual maturity rate defined by Tanner Criteria [23]. ^2^ Classification of BMI/age according to WHO growth curves (2006–2007) [24]. ^3^ Student’s *t*-test. ^4^ Chi-square test.

**Table 2:** Protein levels in the two clusters of DNA damage.

Protein (pM)	Cluster 1 (*n* = 62)	Cluster 2 (*n* = 78)	*p*-Value ^1^
CDK8–CCNC	2265.5 (1989.0; 2468.1)	2399.5 (2093.7; 2708.4)	0.008
KYNU	985.4 (837.7; 1150.2)	1061.8 (906.0; 1207.8)	0.028
PIK3CA–PIK3R1	1332.7 (1132.4; 1523.0)	1424.0 (1228.6; 1614.8)	0.044
PRKCB	24,843.2 (20,265.4; 27,851.2)	25,460.1 (21,616.7; 29,323.1)	0.024

Cluster 1, lower DNA damage, and cluster 2, higher DNA damage. CDK8: Cyclin-dependent kinase 8; CCNC: Cyclin C; KYNU: kynureninase; PIK3CA: phosphatidylinositol 3-kinase catalytic subunit alpha; PIK3R1: phosphatidylinositol 3-kinase regulatory subunit 1; PRKCB: protein kinase C beta. ^1^ ANCOVA, adjusted for age, sex, BMI, and energy intake.

**Table 3:** Statistically significant proteins associated to DNA damage between clusters 1 and 2.

Gene ID	Full Name	Function	Action
CDK8 1024	Cyclindependent kinase 8	This kinase and its regulatory subunit, cyclin C, are components of the mediator transcriptional regulatory complex involved in both transcriptional activation and repression. This kinase phosphorylates the carboxy-terminal domain of RNA polymerase II [32,33,34]	Pro-inflammatory [35,36]
CCNC 892	Cyclin C	Cyclin C interacts with cyclin-dependent kinase 8 and induces the phosphorylation of RNA polymerase II. The level of mRNAs for the gene that encodes this protein reaches its peak in the G1 phase of the cell cycle [32,37]	Anti- and pro-inflammatory [38]
KYNU 8942	Kynureninase	KYNU is a pyridoxal-5′-phosphate-dependent enzyme that catalyzes the cleavage of L-kynurenine and L-3-hydroxykynurenine. KYNU is involved in biosynthesis of NAD cofactors from tryptophan through kynurenine pathway [39].	Pro-inflammatory [39,40]
PIK3CA 5290	Phosphatidylinositol 3-kinase catalytic subunit alpha	PIK3CA is the catalytic subunit of phosphatidylinositol 3-kinase (PI3K). It uses ATP to phosphorylate phosphatidylinositol, phosphatidylinositol-4-phosphate, and phosphatidylinositol 4,5-bisphosphate. The gene that encodes this protein has been found to be oncogenic [41].	Pro-inflammatory [10,41]
PIK3R1 5295	Phosphatidylinositol 3-kinase regulatory subunit 1	PIK3R1 is the regulatory subunit of phosphatidylinositol 3-kinase, which phosphorylates the inositol ring of phosphatidylinositol at the 3-prime position. Phosphatidylinositol 3-kinase plays an important role in metabolic actions of insulin [41].	Pro-inflammatory [10,41]
PRKCB 5579	Protein kinase C beta	PRKCB is a member of protein kinase C (PKC) family. PKC family members phosphorylate a wide variety of protein targets and are involved in diverse cellular signaling pathways. PRKCB has been involved in many different cellular functions, such as B cell activation, apoptosis, endothelial cell proliferation, and intestinal sugar absorption [42].	Pro-inflammatory [43]

Gene Id from National Center of Biotechnology Information in https://www.ncbi.nlm.nih.gov/, accessed on 19 July 2021 [44].

**Table 4:** Association of proteins and DHA and EPA levels with DNA damage.

Protein (pM)	R	R Square	Adjusted R Square	Durbin–Watson	*p*-Value ^1^
All proteins	0.44	0.19	0.13	1.93	0.002
CDK8–CCNC	0.42	0.18	0.13	1.98	0.001
KYNU	0.42	0.18	0.13	1.94	0.001
PIK3CA–PIK3R1	0.41	0.17	0.12	1.98	0.002
PRKCB	0.42	0.18	0.13	1.97	0.001

^1^ Multiple regression analysis. Dependent variable: tail intensity; Predictors: proteins, DHA, EPA, energy intake, BMI, age, and sex.

## Data Availability

The datasets supporting the conclusions of this article are included within the article and its additional files. The raw data are available from the corresponding author on reasonable request.

## Supplementary Materials

The following are available online at https://www.mdpi.com/article/10.3390/nu13082483/s1, Supplementary Table S1.
